# Physiological influences on neurovascular coupling: A systematic review of multimodal imaging approaches and recommendations for future study designs

**DOI:** 10.1113/EP092060

**Published:** 2024-10-11

**Authors:** Joel S. Burma, Damian M. Bailey, Nathan E. Johnson, James K. Griffiths, Josh J. Burkart, Clara A. Soligon, Elizabeth K. S. Fletcher, Raelyn M. Javra, Chantel T. Debert, Kathryn J. Schneider, Jeff F. Dunn, Jonathan D. Smirl

**Affiliations:** ^1^ Cerebrovascular Concussion Laboratory, Faculty of Kinesiology University of Calgary Alberta Canada; ^2^ Sport Injury Prevention Research Centre, Faculty of Kinesiology University of Calgary Calgary Alberta Canada; ^3^ Human Performance Laboratory, Faculty of Kinesiology University of Calgary Calgary Alberta Canada; ^4^ Libin Cardiovascular Institute of Alberta University of Calgary Calgary Alberta Canada; ^5^ Alberta Children's Hospital Research Institute University of Calgary Calgary Alberta Canada; ^6^ Hotchkiss Brain Institute University of Calgary Calgary Alberta Canada; ^7^ Neurovascular Research Laboratory, Faculty of Life Sciences and Education University of South Wales Pontypridd UK; ^8^ Department of Biomedical Engineering, Cumming School of Medicine University of Calgary Calgary Alberta Canada; ^9^ Department of Clinical Neurosciences, Cumming School of Medicine University of Calgary Calgary Alberta Canada; ^10^ Sport Medicine Centre University of Calgary Calgary Alberta Canada; ^11^ Department of Radiology, Cumming School of Medicine University of Calgary Calgary Alberta Canada

**Keywords:** electroencephalography, functional hyperaemia, functional magnetic resonance imaging, multimodal imaging, neurovascular coupling

## Abstract

In this review, we have amalgamated the literature, taking a multimodal neuroimaging approach to quantify the relationship between neuronal firing and haemodynamics during a task paradigm (i.e., neurovascular coupling response), while considering confounding physiological influences. Original research articles that used concurrent neuronal and haemodynamic quantification in humans (*n* ≥ 10) during a task paradigm were included from PubMed, Scopus, Web of Science, EMBASE and PsychINFO. Articles published before 31 July 2023 were considered for eligibility. Rapid screening was completed by the first author. Two authors completed the title/abstract and full‐text screening. Article quality was assessed using a modified version of the National Institutes of Health Quality Assessment Tool for Observational Cohort and Cross‐Sectional Studies. A total of 364 articles were included following title/abstract and full‐text screening. The most common combination was EEG/functional MRI (68.7%), with cognitive (48.1%) and visual (27.5%) tasks being the most common. The majority of studies displayed an absence/minimal control of blood pressure, arterial gas concentrations and/or heart rate (92.9%), and only 1.3% monitored these factors. A minority of studies restricted or collected data pertaining to caffeine (7.4%), exercise (0.8%), food (0.5%), nicotine (2.7%), the menstrual cycle (0.3%) or cardiorespiratory fitness levels (0.5%). The cerebrovasculature is sensitive to numerous factors; thus, to understand the neurovascular coupling response fully, better control for confounding physiological influences of blood pressure and respiratory metrics is imperative during study‐design formulation. Moreover, further work should continue to examine sex‐based differences, the influence of sex steroid hormone concentrations and cardiorespiratory fitness.

## Abstract (highlights)

- **What is the topic of this review?**
  This review examines literature using simultaneous multimodal neuroimaging to measure the relationship between neuronal firing and haemodynamics during task paradigms (i.e., neurovascular coupling). This review focuses on the control of physiological, psychological and lifestyle factors of the included articles.
- **What advances does it highlight?**
  The review underscores the critical need for improved control of physiological and lifestyle confounders, such as blood pressure, carbon dioxide levels, caffeine intake, exercise, menstrual cycle phase and sex comparisons, in study designs to ensure more accurate and reliable findings in neurovascular coupling research.

## 1 INTRODUCTION

**FIGURE 1:**
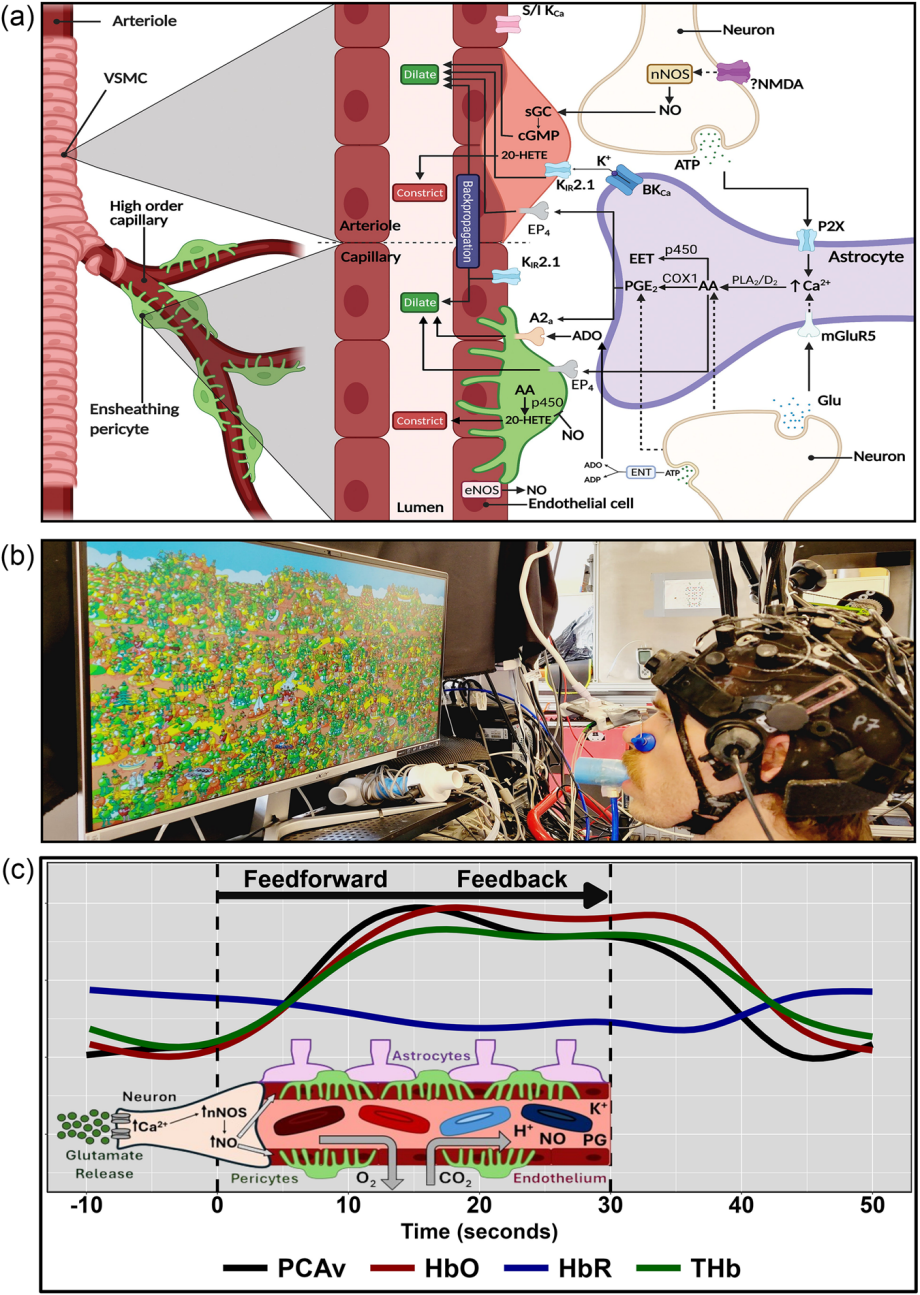
(a) The cerebrovascular tree branching into capillaries and the components of the neurovascular unit. This also denotes the NVC pathways that produce dilatation and constriction of the location vasculature surrounding the neurons, astrocytes and pericytes. Reused with permission from Zhu et al. (2022). (b) An individual completing a visual‐based task. (c) The feedforward and feedback mechanisms underlying the NVC response that correspond to the haemodynamic response seen during a task. It is important to note that these mechanisms are not mutually exclusive at any given time; however, the feedforward mechanism is more pronounced during task onset/offset to achieve the new homeostatic endpoint, whereas the feedback mechanism functions to make fine‐tuning adjustments once this new homeostatic endpoint is achieved. Abbreviations: 20‐HETE, 20‐hydroxyeicosatetraenoic acid; AA, arachidonic acid; ADO, adenosine; BKCa, large‐conductance calcium‐activated potassium channel; cGMP, cyclic guanosine monophosphate; COX1, cyclooxygenase 1; eNOS, endothelial nitric oxide synthase; ENT, ectonucleotidases; EP_4_, prostaglandin E_2_ receptor 4; HbO, oxygenated hemoglobin; HbR, deoxygenated hemoglobin; K_IR_, inward‐rectifying potassium; mGluR5, metabolic glutamate receptor 5; nNOS, neuronal nitric oxide synthase; NVC, neurovascular coupling; PCAv, posterior cerebral artery velocity; PG, prostaglandins; PLA2, phospholipase A2; PLD2, phospholipase D2; S/IKCa, small and intermediate conductance calcium‐activated potassium channel; sGC, soluble guanylate cyclase; THb, total hemoglobin; VSMC, vascular smooth muscle cells.

The neurovascular unit comprises the interactive cellular network responsible for the regulation of cerebral blood flow (CBF) and blood–brain barrier integrity that synergistically preserves neuronal, glial and vascular homeostasis (Kaplan et al., 2020). The neurovascular unit is integral to establishing efficient clearance of carbon dioxide and other waste products while continuously supplying oxygen and glucose, to which the human brain has evolved exquisite sensitivity given its disproportionately high mass‐specific energy demands, limited energy stores and almost exclusive reliance on aerobic metabolism (Bailey, 2019). Specifically, researchers have coined the term neurovascular coupling (NVC; or functional hyperaemia) to describe the haemodynamic response as a function of neuronal activation or inhibition, which functions through feedforward and feedback mechanisms (Figure 1; Iadecola, 2017). The feedforward works through the neuronal signalling of nitric oxide, which affects vasodilatation, whereas the feedback loop is characterized as metabolic by‐products from working cortical tissues resulting in localized vasodilatation (Beishon et al., 2021; Iadecola, 2017).

A large understanding of NVC has come through preclinical models (i.e., experiments completed on animals) because these approaches enable invasive assessments to expose fundamental mechanisms (Gao et al., 2017). These methods have allowed researchers to stimulate specific neurons and quantify the impact of neuronal firing on the localized haemodynamic responses (Iordanova et al., 2015). However, the translational significance for humans remains questionable (Seyhan, 2019). Specific disease progression and drug‐based interventions might therefore be different and not directly transferable to humans (e.g., chronic traumatic encephalography, Alzheimer's disease) (Semple et al., 2013).

The development and progression of neuroimaging modalities have been fruitful in improving our understanding of both the neurovascular unit and the NVC response in humans, given that invasive protocols are not ethical or widely feasible. However, a limitation of these techniques is that previous NVC studies have been quantified largely through the use of indirect haemodynamic responses as a surrogate for neuronal activation (Poplawsky et al., 2021). To counteract this, researchers have begun to use and recommend a multimodal imaging approach that can measure neuronal activation and cerebral haemodynamics concurrently (Calhoun & Sui, 2016; Tulay et al., 2019). A multimodal approach to assessing NVC is superior to an unimodal approach because it provides a comprehensive view of the vascular and neuronal activities of the brain (Tulay et al., 2019; Uludağ & Roebroeck, 2014; Yeung & Chu, 2022). Additionally, a multimodal approach can leverage the strengths of each modality to compensate for the limitations of others.

It is important to consider that the underlying physiology associated with the NVC response is integrative in nature, meaning that numerous variables aside from brain parenchyma will confound the haemodynamic signal (Willie et al., 2011, 2014); these can include physiological, psychological and lifestyle factors. Within the brain, arterial carbon dioxide levels, blood pressure, autonomic nervous system activity and cardiac output influence CBF (Willie et al., 2011, 2014). For example, hypercapnic elevations in the partial pressure of carbon dioxide ($P_{\mathrm{ET},CO_{2}}$) have been shown to elicit ∼3%–6% increases in CBF per millimetre of mercury, and hypocapnia results, leading to ∼1%–3% reductions (Willie et al., 2011, 2014). Likewise, given that the baroreflex is active on a beat‐by‐beat basis, changes in diameter of peripheral arteries will result in blood pressure fluctuations transmitted to the cerebrovascular bed and are present in the recorded haemodynamic signal (Julien, 2006). A benefit of adopting a concurrent multimodal imaging approach is that it minimizes the confounding influence(s) of the aforementioned physiological variables, because slight deviations in blood pressure, respiratory patterns or heart rate will be identical between recordings.

In addition to physiological stimuli, demographic confounders have also been shown to impact cerebrovascular regulation (e.g., age, sex, fitness and hormones) (Kennedy et al., 2022). It is well established that after puberty, females will exhibit elevated global CBF compared with their male counterparts (Alwatban et al., 2021) and that both sexes will experience reductions in cerebral blood velocity after the age of 30 years (∼0.5% reduction per year; Ainslie et al., 2008). Additionally, neuronal atrophy occurs with ageing, leading to a reduced NVC response (Lipecz et al., 2019), with sex potentially modifying age‐related changes (Koep et al., 2023). Nevertheless, regular exercise has demonstrated that these neuronal and vascular deficits can be attenuated (Ainslie et al., 2008; Ahlskog et al., 2011; Bailey et al., 2013), potentially leading to more efficient NVC. Finally, lifestyle habits [e.g., caffeine consumption (Addicott et al., 2009), acute exercise (Burma, Copeland, et al., 2020, Burma, Macaulay, et al., 2020; Burma et al., 2021) and tobacco use (Toda & Okamura, 2016)] are also known to impact cerebrovascular function, with few investigations exploring the direct impact that these habits have on the NVC response.

These factors are capable of being controlled during the study design (e.g., dietary control/restriction, participant matching and physiological monitoring) and/or statistical analysis (e.g., modelling and stratification) within a study. This helps to ensure internal validity, in that the neuronal task is the primary variable eliciting a haemodynamic response. The purpose of this systematic review was to amalgamate the literature that has adopted a multimodal imaging approach consisting of at least one neuronal and haemodynamic concurrent assessment in humans during a task that would challenge the cortices (e.g., nBack, finger tapping or Stroop). These articles are critiqued based on the control that these studies used for physiological influences (e.g., blood pressure and carbon dioxide levels), demographic characteristics (e.g., sex, age, hormones and fitness) and lifestyle habits (e.g., acute exercise and caffeine and alcohol consumption). Additionally, this review seeks to identify those clinical populations in which multimodal NVC assessments have been conducted. Given that multimodal imaging is becoming more commonplace within the broader research fields, this review will be paramount to assist researchers in developing high‐quality study designs across the field of neuroimaging, with specific control for confounding factors.

## 2 MATERIALS AND METHODS

### 2.1 Protocol, registration and purpose of review

An a priori search was completed in the Prospero database to identify whether the topic of the current review had been completed previously. No registered protocols were found, hence the present systematic review was registered as a novel research question (ID: CRD42023418405). This review was completed in accordance with the standards and recommendations set forth by the Preferred Reporting Items for Systematic Reviews and Meta‐Analysis (PRISMA) guidelines, excluding items 12 (effect measures), 15 (certainty assessment) and 21 (certainty of evidence) (Liberati et al., 2009; Moher et al., 2009). These were unable to be completed owing to the present review focusing on the methodological design of the included articles, rather than the results.

### 2.2 Information sources

The search terms and strategy were developed by J.S.B. and J.D.S., which were refined for approval by C.T.D., K.J.S. and J.F.D. Likewise, J.S.B. and J.D.S. created the inclusion and exclusion criteria, which were checked and refined by the same authors. The finalized search strategy, shown in Table 1, was adapted for the following databases: PubMed, Scopus, Web of Science, EMBASE and PsychINFO. These databases index the majority of all physiologically based articles. All articles were downloaded, then uploaded into an Endnote (Philadelphia, PA, USA) library that was used for reference management. Duplicates were removed.

**TABLE 1:** Search concepts/terms used to identify potential articles for inclusion.

Concept	Terms
Neural	EEG, qEEG, ERP, electroencephalo*, neuroelectric, event‐related potential, magnetoencephalo*, neurophys*, electrocortico*, evoked potential
Haemodynamic	fNIRS, near‐infrared spectroscopy, NIRS, haemodynami*, cerebral oxyg*, transcranial Doppler, transcranial color, TCD, TCCD, cerebral blood flow velocity, cerebral blood velocity, arterial blood velocit*, middle cerebral artery, posterior cerebral artery, magnetic resonance imag*, arterial spin label*, positron emission tomoraph*, microvascula*, BOLD, blood oxygen, blood‐oxygen, hemoglobin, PET, MRI
Neurovascular coupling	Coupling, neurovascular unit, functional hyperemia, neural‐hemodynamic*, neural hemodynamic*, functional‐hyperemia, cerebral hyperemia, brain hyperemia, cerebral hyperaemia, brain hypaeremia, cerebral‐hyperemia, brain‐hyperemia, cerebral‐hyperaemia, brain‐hyperaemia, brain coupling, brain‐coupling, hyperaemia demand
Task	Task, paradigm, sequence, protocol, exercise, engagement, visual, auditory, cognitive, motor, somatosensory

Abbreviations: BOLD, blood‐oxygen‐level‐dependent; ERP, event‐related potential; fNIRS, functional near‐infrared spectroscopy; NIRS, near‐infrared spectroscopy; PET, positron emission tomography; qEEG, quantitative electroencephalography; TCCD, transcranial colour Doppler; TCD, transcranial Doppler.

### 2.3 Eligibility criteria

Studies were included if the authors took a multimodal imaging approach that consisted of an assessment of both neuronal activation and haemodynamic measures simultaneously. Neuronal imaging devices included EEG (scalp and intracranial), magnetoencephalography or microelectrode arrays. Haemodynamic techniques included functional MRI (fMRI), functional near‐infrared spectroscopy (fNIRS), transcranial Doppler ultrasound (TCD), positron emission tomography, single‐photon emission computed tomography, arterial spin labelling and diffuse optical tomography. Articles before 31 July 2023 were assessed for eligibility. Investigations that completed both neuronal and haemodynamic assessments in separate testing sessions were excluded because they were not completed simultaneously. This was justified based on the fact that non‐concurrent assessments would be more influenced by physiological differences between testing sessions. For example, if EEG data are collected in an upright position in a quiet room, whereas an fMRI scan is completed in a noisy scanner in a supine position, the difference in posture and environmental stimuli might change respiration rate, blood pressure or heart rate. Conversely, with a concurrent approach, an increase/decrease in heart rate would occur in all recorded traces.

Studies had to be completed in humans that were original research articles (e.g., cross‐sectional, cohort or pre‐experimental), with ≥10 participants during a task paradigm. A minimum of 10 participants was chosen because case reports and studies with few participants often suffer from greater biases and methodological flaws, reducing the reliability and generalizability of their findings. A task was defined as a non‐resting‐state assessment, whereby participants had to engage in a task designed to increase activation in a specific cortical region. Therefore, non‐original articles were excluded (e.g., review articles, dissertations, theses, commentaries, opinion pieces, conference proceedings or book chapters), in addition to case studies and series completed in nine or fewer participants. Resting‐state investigations while awake and/or while sleeping were not included. Studies that contained animals only were excluded, but if a study contained both animal and human results, the human results were included in the review. Finally, the full text had to be written in English to be included in the present review.

### 2.4 Study selection

Rayyan (https://rayyan.ai/), a systematic review software platform (Ouzzani et al., 2016), was used for the title and abstract screening and the full‐text screening. J.S.B. completed a rapid screening to exclude articles without abstracts, non‐original research and/or animal studies. For the title and abstract and full‐text screening, a blinded approach was taken, whereby two authors voted on each article. This screening was completed by N.E.J., J.B., E.F. and C.A.S. as reviewer 1, and with J.S.B. as reviewer 2. Any conflicts were settled by a discussion between the authors. During the title and abstract screening, articles were excluded if an article did not meet the aforementioned eligibility criteria, without the authors stating the specific exclusion reason. However, during the full‐text review, reviewers had to state the reason for exclusion for each article not included. An a priori hierarchy was developed to minimize reviewer conflicts, which consisted of the following: (1) wrong outcome (e.g., study using only one imaging modality, study using a multimodal protocol that was not conducted concurrently); (2) wrong study design (e.g., case study with <10 participants, review article or conference proceeding); (3) wrong population (e.g., concurrent multimodal imaging study conducted in animals); and (4) wrong language (e.g., full text not written in English).

### 2.5 Risk of bias

Risk‐of‐bias assessment was completed on all articles using a modified version of the National Institutes of Health Quality Assessment Tool for Observational Cohort and Cross‐Sectional Studies (Health, 2014). Given that a major aim of the review was to identify the control within the included studies, an additional four questions were added:

1. Did the study control for heart rate, blood pressure, breathing patterns and/or carbon dioxide levels?
2. If the study included female participants, did they measure hormones/menstrual cycle in any capacity?
3. Did the study measure and/or control cardiorespiratory fitness?
4. Did the study control/restrict caffeine, alcohol exercise, and/or smoking for ≥6 h before study commencement?

From these questions, reviewers were required to categorize the study design of the articles into one of the following three domains: (1) poor (high risk of bias, with minimal control for covariates); (2) fair (moderate risk of bias, with some control for covariates); or (3) good (low risk of bias, with adequate control for covariates). It is important to note that the present review is only assessing the control within the study designs of the included study. This does not mean that the results or interpretations of the studies are invalid, because there could have been no difference between populations and/or physiological responses; it simply means that certain aspects were not measured. Likewise, an article that is rated as good might not mean that it is highly valid externally. For example, a study could include a sample of 10 male endurance athletes who completed a NVC task with blood pressure and capnography collected. Given that no females were included, this somewhat minimizes the influence of sex hormones; however, these results would be generalizable only to a specific population. Independent screening was completed with N.E.J., J.G., J.B., C.S. and R.J., as reviewer 1 and with J.S.B. as reviewer 2. Conflicts were settled by a discussion between the authors.

### 2.6 Data extraction

The purpose of the present review was primarily to focus on the methods of the included studies; therefore, the information extracted included the title, author list, published year, country data, sample size, participant demographics (age and sex), including clinical status, haemodynamic technique and region measured, neurophysiological technique and region measured, other physiological measures collected and task(s) undertaken. Data extractions were completed by J.S.B., N.E.J., J.B., J.K.G. and C.A.S.

### 2.7 Planned method of analysis

Different analytical approaches to process multimodal data have been discussed extensively in other reviews (e.g., data‐driven fusion, model‐driven fusion and asymmetric constrained; Calhoun & Sui, 2016; Kalamkar & Geetha, 2023; Tulay et al., 2019; Uludağ & Roebroeck, 2014; Zhang et al., 2020). Therefore, although these approaches will be discussed briefly, the primary purpose of the present review was as follows: (1) to amalgamate the literature and identify the most commonly used multimodal techniques; (2) to assess the extent to which the included studies incorporated some degree of control for covariate and/or modifying influences (e.g., physiological, demographic or daily habits); (3) to characterize the demographics of the included participants across these studies; and (4) to understand the clinical populations in which these techniques have been conducted. It is expected that the methodological approaches of the studies will be heterogeneous regarding the combination of imaging modalities, regions of measurement, task(s) paradigm(s) used, type of statistical analysis and sample participants. This limits the ability to derive any meaningful results and/or meta‐analysis across studies. Therefore, in the present review we aimed to compare and contrast the methods of the included studies, with the goal of guiding future high‐quality multimodal study designs, specifically with the ability to control known covariates. The suggestions will help to enhance the internal validity of subsequent original research investigations.

## 3 RESULTS

### 3.1 Search results

**FIGURE 2:**
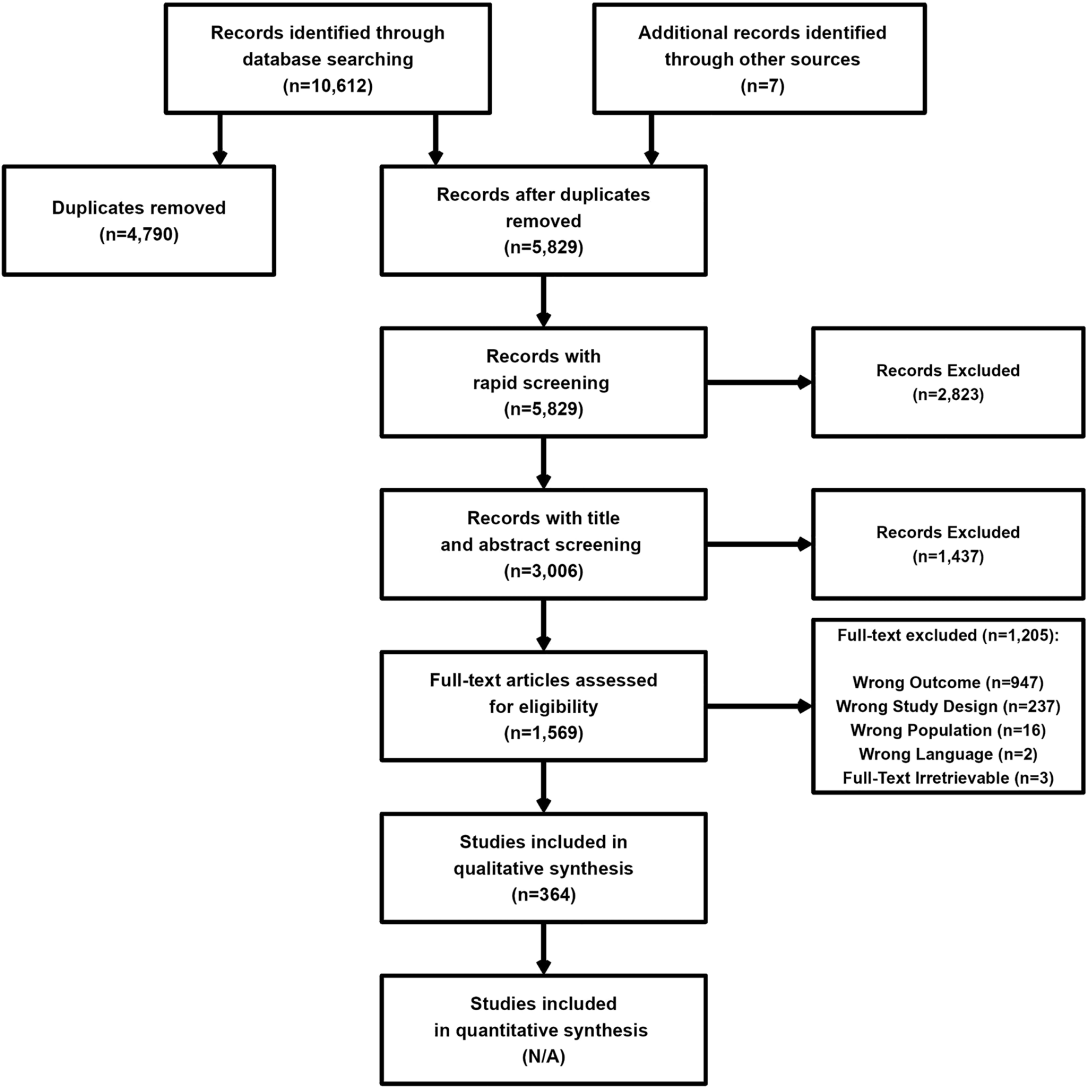
A PRISMA flowchart of literature search and selection. Abbreviation: PRISMA, preferred reporting items for systematic reviews and meta‐analysis.

A total of 10 612 citations were identified across all databases, with an additional seven found through other sources (Figure 2). From these, 4790 were identified as duplicates, leaving 5829 articles that were screened rapidly (Figure 2). Title and abstract screening were completed on 3006 articles, with 1437 articles being excluded at this stage (Figure 2). Full‐text screening assessed the eligibility of 1569 articles, of which 947 were excluded owing to the studies not using a simultaneous neural and haemodynamic quantification, 237 owing to study design (e.g., case study or case series), 16 owing to the population (i.e., animals), 2 owing to a language other than English, and 3 owing to the full‐text not being retrievable (Figure 2). Therefore, data extraction and risk‐of‐bias assessments were completed on a total of 364 articles (Figure 2), highlighting an attrition of ∼97%.

### 3.2 Study demographics and characteristics

**FIGURE 3:**
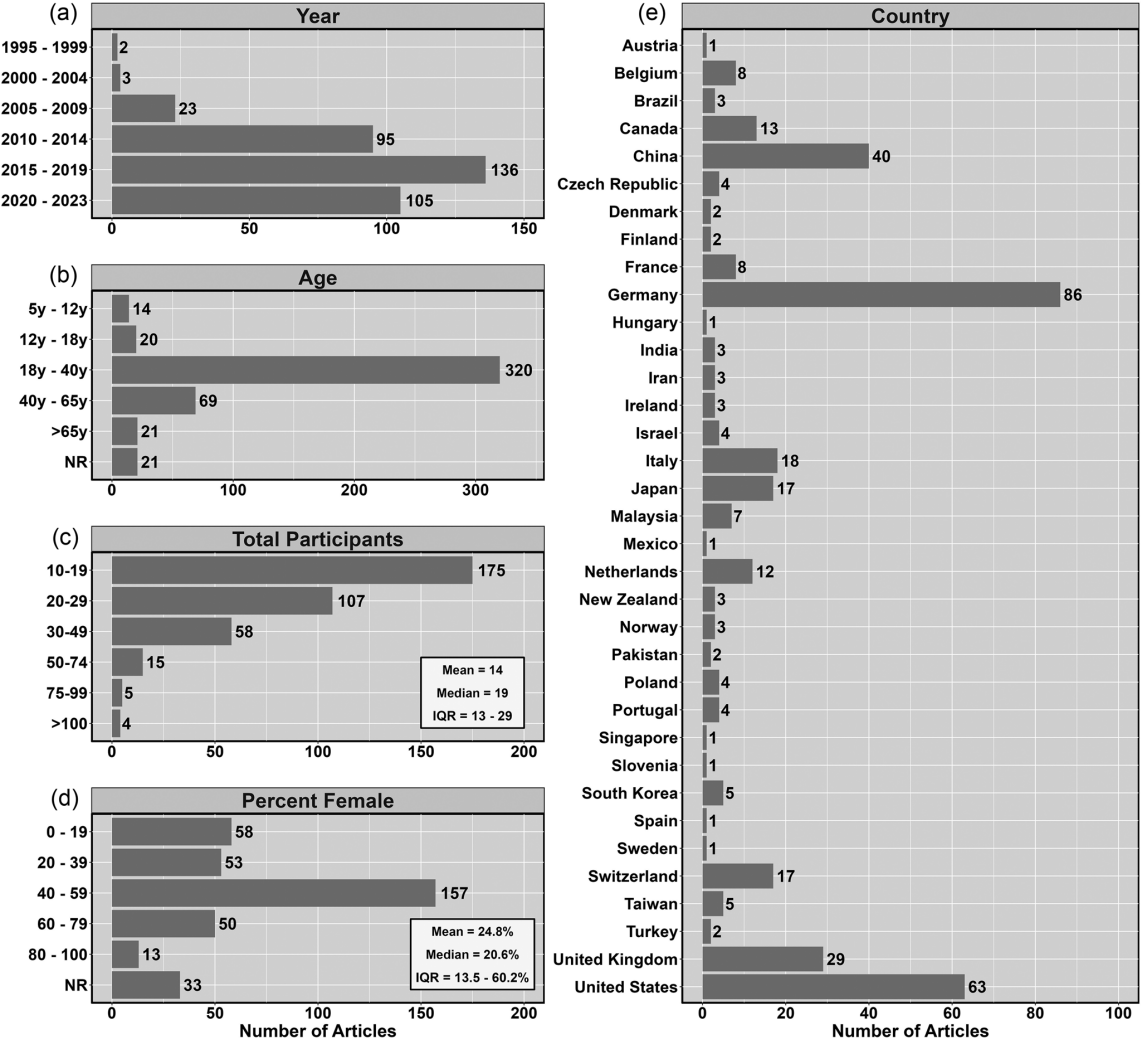
Demographic characteristics of the 364 included studies based on: (a) year published; (b) age of included participants; (c) total number of participants; (d) proportion of female participants; and (e) country where data collection occurred. Note that some studies had more than one age group and/or were conducted in more than one study; therefore, this will exceed the total number of studies. Abbreviation: NR, not reported.

**FIGURE 4:**
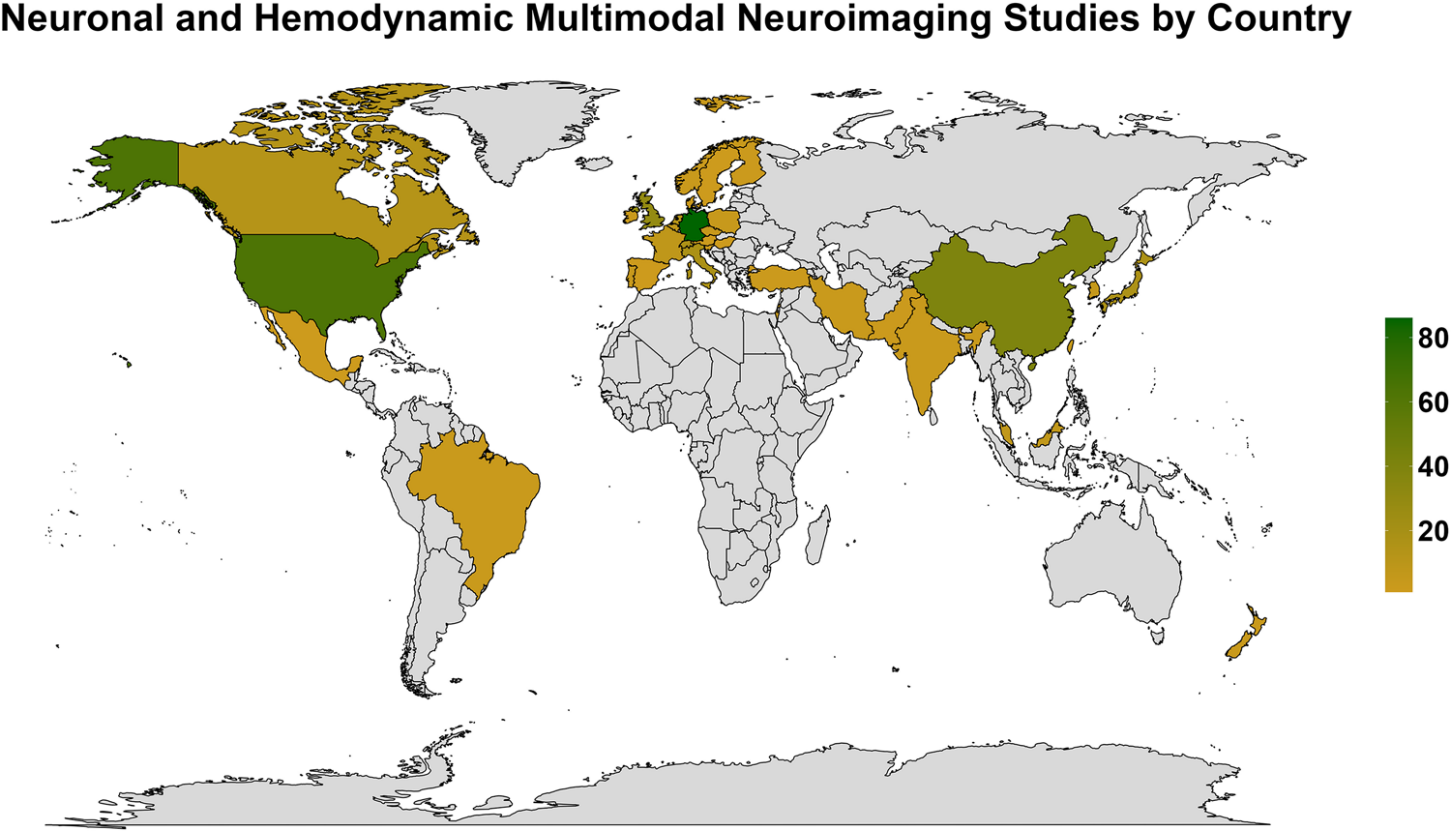
A heat map displaying the number of multimodal studies included from each country that carried out a concurrent assessment of neuronal and haemodynamic function. Countries with no studies are displayed in grey.

Figure 3 illustrates the characteristics of the included studies, with the majority being completed in North America and/or Europe (*n* = 271 of 364, 74.5%). Figure 4 shows a heat map of the location of the included citations. The first included study was in 1995, with research increasing exponentially each decade (Figure 3). The average number of participants included in the studies was 24.8 (median 20.0, interquartile range 15.0–27.3; Figure 3). The average female representation was 42.6% across all studies (median 47.4%, interquartile range 34.5%–60.2%), with 9.1% (*n* = 33) of articles not reporting or defining the sex of the participants (Figure 3). Younger adulthood was the most studied population (87.9%; *n* = 324), followed by middle adulthood (19.0%; *n* = 69) (Figure 3).

**FIGURE 5:**
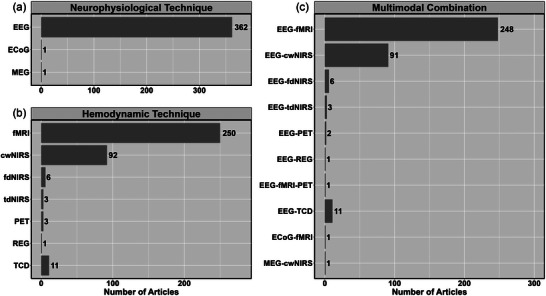
The neuroimaging techniques used in the 364 studies broken down as follows: (a) neurophysiological technique; (b) haemodynamic technique; and (c) multimodal combination of the neurophysiological and haemodynamic techniques. Abbreviations: cwNIRS, continuous‐wave near‐infrared spectroscopy; ECoG, electrocorticography; fdNIRS, frequency‐domain near‐infrared spectroscopy; fMRI, functional magnetic resonance imaging; MEG, magnetoencephalography; PET, positron emission technology; REG, rheoencephalography; TCD, transcranial Doppler ultrasound; tdNIRS, time‐domain near‐infrared spectroscopy.

**FIGURE 6:**
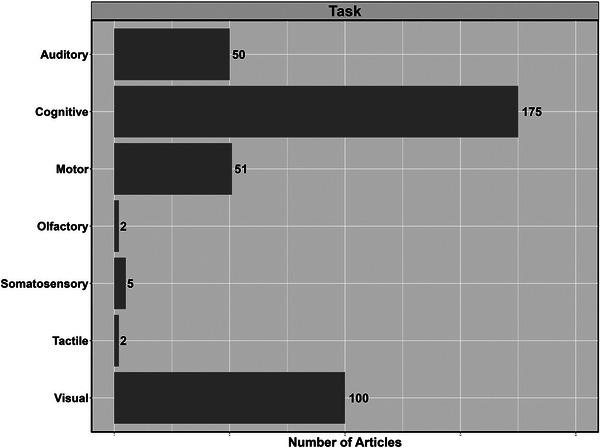
Tasks included across studies broken down into domains that challenge different portions of the cerebral cortices. Note that some studies might have included more than one task, hence the total number will exceed the number of studies included in the review.

**FIGURE 7:**
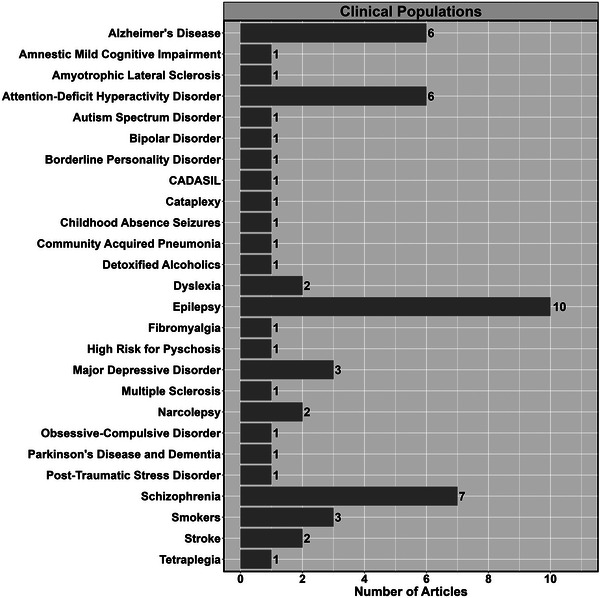
The number of multimodal neuroimaging assessments of clinical populations that have been completed during task activation. Abbreviation: CADASIL, cerebral autosomal dominant arteriopathy with subcortical infarcts and leukoencephalopathy.

The most common neurophysiological techniques used were EEG (99.4%; *n* = 362), with only one study incorporating electrocorticography (0.3%) and magnetoencephalography (0.3%), respectively (Figure 5). The most common haemodynamic techniques used were fMRI (68.7%; *n* = 250), continuous‐wave NIRS (25.2%; *n* = 91) and TCD (3.0%; *n* = 11) (Figure 4). The most commonly used tasks fell into domains of cognitive (48.1%, *n* = 175), visual (27.5%, *n* = 100), motor (14.0%, *n* = 51) and auditory (13.7%, *n* = 50) (Figure 6). Figure 7 demonstrates the different clinical populations in which multimodal approaches have been used. The most common populations included epilepsy (*n* = 10), schizophrenia (*n* = 7), Alzheimer's disease (*n* = 6) and attention‐deficit hyperactivity disorder (*n* = 6) (Figure 7).

### 3.3 Physiological control and risk of bias

**FIGURE 8:**
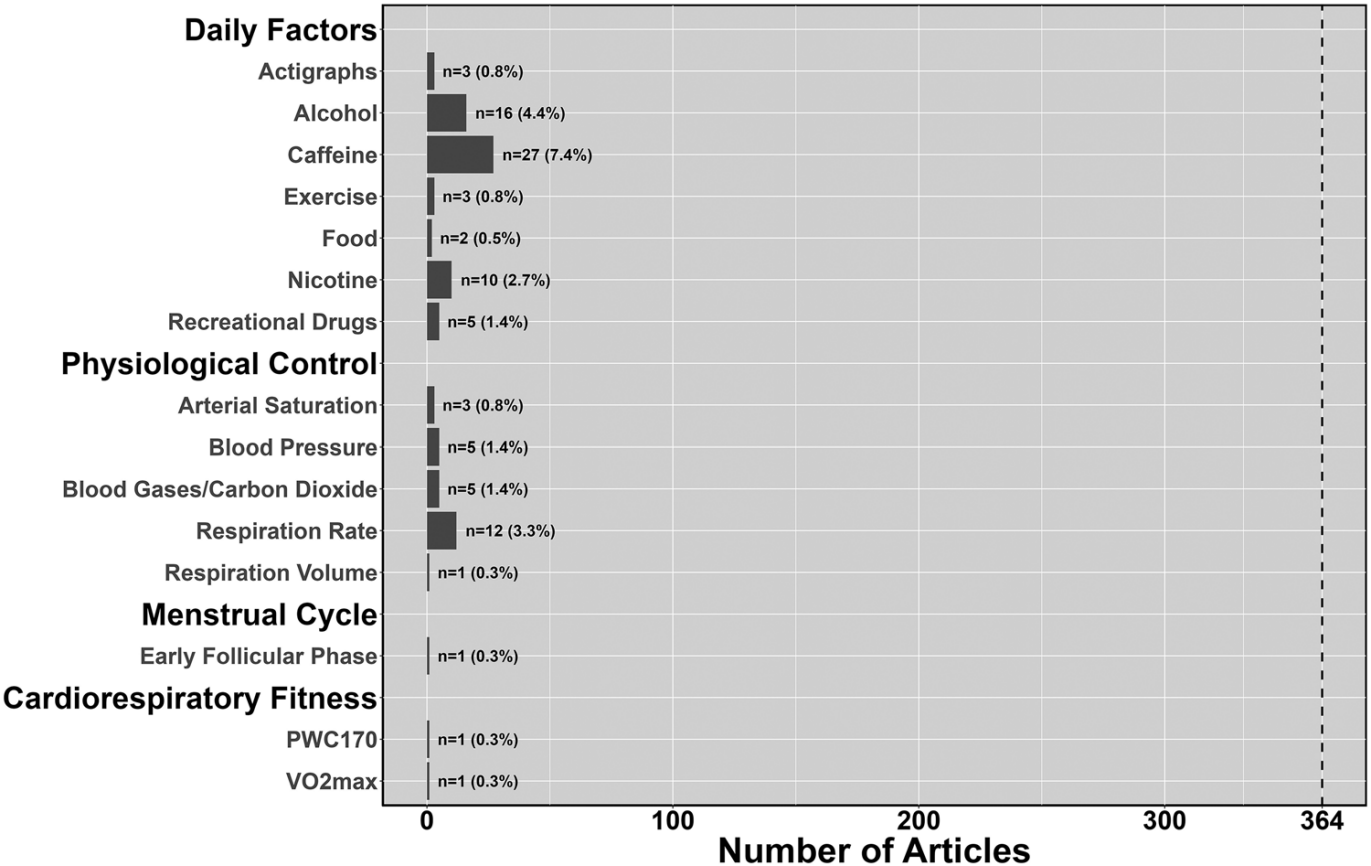
The number and total percentage of articles that used physiological control in their study design based on daily factors, physiological variables, menstrual cycle considerations and cardiorespiratory fitness levels.

Physiological measures were collected in a total of 5.2% (*n* = 19) of studies, including respiratory rate (3.3%, *n* = 12), blood pressure (1.4%; *n* = 5), $P_{\mathrm{ET},CO_{2}}$ (1.4%; *n* = 5) and/or pulse oximetry (0.8%; *n* = 3) (Figure 8). Only one article (0.3%) controlled the menstrual cycle phase or hormones in any capacity, whereby the included females were tested during the early follicular phase (Figure 8). Only two articles (0.5%) measured cardiorespiratory fitness, with one of these using a maximal oxygen uptake test and the other using the Physical Working Capacity 170 (PWC‐170) test (Figure 8). Finally, a small number of studies restricted and/or controlled for alcohol (4.4%, *n* = 16), caffeine (7.4%, *n* = 27), exercise (0.8%, *n* = 3), food (0.5%, *n* = 2) and/or nicotine (2.7%, *n* = 10) (Figure 8). Owing to the general lack of physiological control across studies, only 1.37% (*n* = 5) were rated as good and with a low risk of physiological bias owing to adequate control, 5.8% (*n* = 21) were rated as fair and with a moderate risk of physiological bias owing to inadequate control, and 92.9% (*n* = 338) were rated as poor and with a high risk of physiological bias owing to a complete lack of control of covarying factors (Table 2).

**TABLE 2:** Risk‐of‐bias assessments for the included articles regarding the physiological control used in the methodology.

	Poor	Fair	Good	Total
Neuronal				
EEG	336 (92.8%)	21 (5.8%)	5 (1.4%)	362
ECoG	1 (100%)	0 (0%)	0 (0%)	1
MEG	1 (100%)	0 (0%)	0 (0%)	1
Haemodynamics				
fMRI	234 (93.6%)	15 (6%)	1 (0.4%)	250
cwNIRS	87 (94.6%)	4 (4.3%)	1 (1.1%)	92
fdNIRS	6 (85.7%)	1 (14.3%)	0 (0%)	7
tdNIRS	3 (100%)	0 (0%)	0 (0%)	3
PET	3 (100%)	0 (0%)	0 (0%)	3
REG	1 (100%)	0 (0%)	0 (0%)	1
TCD	7 (63.6%)	1 (9.1%)	3 (27.3%)	11

Abbreviations: cwNRIS, continuous wave functional near‐infrared spectroscopy; ECoG, electrocorticography; fdNRIS, frequency‐domain functional near‐infrared spectroscopy; fMRI, functional magnetic resonance imaging; MEG, magnetoencephalography; PET, positron emission tomography; REG, rheoencephalography; TCD, transcranial Doppler ultrasound; tdNRIS, time‐domain functional near‐infrared spectroscopy.

## 4 DISCUSSION

The main aims of the present review were to identify the number of articles that have used multimodal imaging approaches to assess NVC and perform a quality assessment regarding the study design of the included articles on their control for physiological, demographic and daily habit confounding influences. The majority of articles had an absence or minimal control for the aforementioned factors (∼93%). This does not mean intrinsically that these studies are not internally valid owing to uncontrolled/measured covariates. Instead, it highlights the need for larger‐scale investigations that seek to control for as many covariates as possible during the study design and statistical analysis. Recommendations and guidelines to increase the internal validity of multimodal studies will be discussed to help guide and inform future high‐quality investigations (Table 3). Of note, a dearth of clinical investigations was also noticed, perhaps explaining the uncertainty about whether NVC dysfunction is a cause or merely a consequence of underlying CNS pathology. Likewise, a lack of sex‐based comparisons was noted, which becomes more imperative to investigation clinically, because disease progression, treatment and recovery are known to differ by sex for stroke, dementia, multiple sclerosis, traumatic brain injury and other neurological conditions (Appelros et al., 2009; Bazarian et al., 2010; Coyle, 2021; Mielke, 2018; Mollayeva et al., 2018; Vyas et al., 2021).

**TABLE 3:** Practical solutions and technological developments required to enhanced the validity of multimodal neurovascular coupling investigations.

Practical solutions
(1) Where possible, implement monitoring for systemic variables, including blood pressure, blood gases, respiratory rate and heart rate.
(2) The equal inclusion of female participants, with testing happening during days 3–10 of the follicular phase and/or the collection of menstrual cycle and contraceptive usage information.
(3) Participants should avoid caffeine, alcohol, tobacco, vaping and exercise for 12 h prior to testing or the morning before each testing visit.
(4) The use of field‐relevant questionnaires could enhance the ability to include confounding variables in statistical analyses. These could include mental health, previous health history, sleep and fatigue.
(5) Reporting effects sizes in the results section or as supplemental material to help understand the required sample sizes for subsequent studies.
Technological developments
(1) Enhance access to metal‐free, non‐invasive blood pressure monitoring devices to be used within a magnetic resonance scanner.
(2) Develop more end‐tidal monitoring solutions to allow for the concurrent assessment of carbon dioxide and oxygen values.
(3) Create valid, cost‐effective hormonal profile analyses to allow for the quantification of hormonal levels at the start of testing sessions.

### 4.1 Influence of systemic cardiorespiratory factors on the cerebrovasculature

Neuronal activity is only one factor that influences the cerebrovasculature (Willie et al., 2014). Systemic influences of both blood pressure and carbon dioxide are well known to have prominent impacts on CBF regulation (Brassard et al., 2021; Claassen et al., 2021). Slight changes in blood pressure will lead to changes in CBF (Smith & Ainslie, 2017). It is important to note that these factors are not stationary during NVC assessments. For example, highly trained individuals can exhibit pronounced Mayer wave activity occurring at ∼0.10 Hz, which confounds the ability to extract brain activity data independently (see Burma, Wassmuth, Kennedy, et al., 2021: fig. 6). Furthermore, blood pressure will be adjusted on a beat‐to‐beat basis owing to myogenic influences, also known as the Bayliss effect (Blum et al., 1999; Brassard et al., 2023). This is especially prominent in small arterioles, where to avoid over‐ and underperfusion, calcium signalling leads to constriction or dilatation of the endothelial smooth muscles, respectively (Brozovich et al., 2016). Owing to these inherent myogenic and sympathetic influences on blood pressure, it is imperative that future NVC studies should monitor and control for these factors (Claassen et al., 2021). As mentioned above, elevations and reductions in $P_{\mathrm{ET},CO_{2}}$ will elicit ∼3%–6% increases and ∼1%–3% reductions in CBF per millimetre of mercury, respectively (Willie et al., 2011, 2014). Previous work has denoted that the NVC response is altered during hypercapnic and hypocapnic states (Maggio et al., 2014). Therefore, seemingly mild increases/decreases in $P_{\mathrm{ET},CO_{2}}$ can have a large impact on overall NVC metrics (Maggio et al., 2014). For example, in comparison to healthy controls, a clinical population might be more nervous about completing a multimodal study, especially if one occurs within a noisy MRI scanner and/or with a lot of equipment. This might result in participants artificially hyperventilating, causing a slight vasoconstrictive/sympathetic response, ultimately producing artificially underestimated NVC metrics (Smith & Ainslie, 2017). Finally, researchers need to consider a multitude of potentially confounding physiological factors, including, albeit not exclusively confined to, sex hormones, temperature and stress (Cote et al., 2021; Graff‐Guerrero et al., 2008; Hoiland et al., 2016; Rodriguez et al., 1988; Shin et al., 2021; Stoquart‐ElSankari et al., 2007; Trangmar & González‐Alonso, 2019).

Hence, for a full understanding of the NVC response from brain activity independently, it is imperative that physiological factors are accounted for within the study design (Minati et al., 2011). This can occur in numerous ways; however, the most practical would be to quantify respiratory and cardiovascular parameters concurrently and use these data to remove systemic noise from the recorded haemodynamic data (Caballero‐Gaudes & Reynolds, 2017). Several of the included studies stated that they processed their data within specific frequency bands (e.g., 0.02–0.08 Hz) and/or applied low‐pass filters to minimize the influence of respiration rates (∼0.25–0.33 Hz) and Mayer wave activity (∼0.10 Hz). Moreover, band analysis assumes stationarity within physiological signals, which might not always occur. For example, a respiration rate lower than 9 breaths/min would fall underneath the lower limit of the defined respiratory range, and thus would still confound the data.

Nevertheless, although regression analysis helps to a degree, being able to regress baroreflex activity and respiratory sinus arrythmias out of the haemodynamic waveforms will produce more robust data. This will provide researchers with the ability to examine cerebrovascular influences that occur over a wider frequency range (e.g., 0.01–0.20 Hz). Additionally, specialized equipment can also be used to control for physiological confounders. An example is dynamic end‐tidal forcing technology, allowing for the precise clamping of $P_{\mathrm{ET},CO_{2}}$ on a breath‐by‐breath basis to maintain eucapnia (Fierstra et al., 2013). Despite the well‐documented physiological influences described above, Figure 8 demonstrates that few articles have measured blood pressure and/or capnography concurrently.

Finally, task selection can help to minimize the influence of physiological confounding variables. For example, a complex visual scene search has been shown to lead to a ∼25%–30% increase in posterior cerebral artery velocity compared with reading or viewing moving shapes, which led to an increase of ∼10%–15% (Smirl et al., 2016). Therefore, a task that elicits a more pronounced haemodynamic response will help to ensure that this attenuates the influence of blood pressure or $P_{\mathrm{ET},CO_{2}}$ changes and optimize the ability to ‘force’ a physiological signal out of the baseline ‘noise’ to improve detection sensitivity. Moreover, related research has explored how the number of trials undertaken impacts the validity and reliability of the TCD‐derived haemodynamic response (Burma et al., 2022). This is comparable to event‐related potential analysis, which includes multiple trials to isolate the response of the brain to specific events by averaging out random noise from ongoing EEG activity (Beres, 2017). This repetition helps to reduce the aforementioned variability in responses owing to extraneous factors (e.g., attention, fatigue and movement), which provides clearer and more consistent measures (Woodman, 2010). By increasing the number of trials, researchers enhance the statistical power and reliability of their findings, ensuring more accurate interpretations (Woodman, 2010).

*Recommendation 1*: Where feasible, researchers should seek to expand their current protocols/methods to quantify blood pressure and respiration patterns concurrently in order to regress systemic noise out of the haemodynamic data. Additionally, ensuring that sufficient NVC task trials are completed maximizes the physiological signal‐to‐noise ratio.

### 4.2 Sex hormones

It is well documented that sex hormones have an impact on CBF/velocity, whereby elevations in oestrogen, progesterone and testosterone will all result in vasodilatation (Krause et al., 2006). This occurs primarily through the nitric oxide pathways, leading to less calcium uptake within the sarcoplasmic reticulum of the endothelial smooth muscles, hence an absence of actin–myosin cross‐bridging (Brozovich et al., 2016; McNeill et al., 2002). This leads to a relaxation and a dilatory response of the cerebrovasculature (Brozovich et al., 2016). Despite this understanding, a paucity of investigations has occurred into how sex hormones impact the NVC response in both female and male participants. Specifically, previous research investigations have largely cited the unknown influence of hormonal fluctuations associated with the menstrual cycle as a justification for excluding females (Liu & Mager, 2016). However, as stated above, despite this exclusion, there has been a lack of investigations to explore the impact of hormonal fluctuation on cerebrovascular regulation. Previous work has denoted that CBF is highest during the luteal phase, when oestrogen and progesterone levels are highest. It is not known whether the dilatation during this phase will also translate to alterations in the NVC response or whether the cerebrovasculature will maintain its regulation despite a different basal level (Peltonen et al., 2016). Additionally, hormonal fluctuations are complicated further by the use of contraceptive devices (e.g., oral contraceptive pills or intrauterine devices), which can function to prevent the luteinizing hormone surge associated with ovulation and/or cease monthly bleeding (Concas et al., 2022). Finally, a further complication of menstrual cycle research is the heterogeneous cycle lengths that females experience (Bull et al., 2019). A recent review found that only ∼13% of females experience a typical 28 day cycle, hence future research into this domain should be cognizant of inter‐individual variability (Bull et al., 2019).

Historically, the most common approaches to control for alterations in hormones have been by limiting analysis to males or by limiting testing in females during days 3–10 of the follicular phase (Stanhewicz & Wong, 2020). However, females represent >50% of the population, hence it is important that they are included in all research investigations, aside from certain conditions only affecting males (e.g., testicular cancer), to improve generalizability of findings. This has led to point–counterpoint series discussing how best to approach testing of females with varying levels of sex hormones (Stanhewicz & Wong, 2020; Wenner & Stachenfeld, 2020). Wenner and Stachenfeld (2020) argue for the control of the menstrual cycle to maximize the internal validity of a given study design. Conversely, Stanhewicz and Wong (2020) contend that vascular studies should not control for the menstrual cycle in studies that are not designed to address questions about sex steroid hormones (Stanhewicz & Wong, 2020). Although this reduces internal validity, it improves the generalizability of research findings to the general population. For an in‐depth discussion on this topic, readers are referred to publications elsewhere (Giersch et al., 2020; Lindsey, Usselman, Ripplinger, et al., 2023; Lindsey, Kleinbongard, Kassiri, et al., 2023).

Although expensive, the most robust approach to account for hormonal variation would be to monitor hormone variability with fluid markers in larger sample sizes. Hormone concentrations could then be obtained and included within statistical (covariate) models to minimize the influence that this would have on the exposure–outcome relationship of interest. This would allow for females to be included across all menstrual cycle phases, leading to high internal and external validity. Another solution would be to collect data pertaining to cycle day and the use of hormonal contraceptives to enable researchers to stratify their data based on these factors.

Previous studies have focused on biological sex differences rather than self‐identified gender owing to the strong correlation (∼99.7%) between the two (Statistics Canada, 2022), which leads to multicollinearity when both are included in statistical models (Vatcheva et al., 2016). Given that sex differences begin in utero and influence neurological and cerebrovascular systems before birth, it is suggested that sex‐based differences be prioritized when only one variable can be included (Wheelock et al., 2019). Exploring gender identity physiologically would require targeted recruitment of non‐cisgender individuals and careful consideration of confounding factors, such as hormone treatments and mental health (Patino & Ferreira, 2018).

*Recommendation 2*: Research investigations should be designed and powered to include biological sex‐based comparisons through modelling or stratification. Further investigation into the specific influence that sex steroid hormones have on cerebrovascular regulation is rudimentary to understand their potential modularity roles and should be included where possible.

### 4.3 Lifestyle factors

Daily habits, such as acutely consuming caffeine (Addicott et al., 2009; Meno et al., 2005; Pelligrino et al., 2010), alcohol (Balogh et al., 2022; Luchtmann et al., 2013) or food (van Baak, 2008); smoking/vaping (Boms et al., 2010; Toda & Okamura, 2016); and engaging in acute exercise (Burma, Copeland, et al., 2020, Burma, Macaulay, et al., 2020, Burma, Macaulay, Copeland, et al., 2021), are known to impact the cerebrovasculature directly and/or indirectly. Despite the widespread knowledge that these factors will influence the NVC response and endothelial function, Figure 8 demonstrates that relatively few articles took these into consideration when developing their study designs.

Caffeine is an adenosine antagonist, meaning that it will block the vasodilatory effect of adenosine, which, in turn, results in a vasoconstrictive response (Ribeiro & Sebastião, 2010). Furthermore, caffeine will also have an effect on the sympathetic nervous system, promoting the release of catecholamines, such as noradrenaline and adrenaline, which will result in the contraction of the endothelial smooth muscles (Echeverri et al., 2010). Nevertheless, the body routinely functions to ensure that it remains in a homeostatic state. In regular caffeine consumers, the cerebrovasculature will remain in a slightly dilated state, because the expected daily caffeine will constrict the vasculature to normal levels (Addicott et al., 2009). Although it is known that caffeine stimulates this vasoconstrictive response, the impact that this has on the acute signalling of the NVC response is not fully understood. Some researchers will argue against restricting caffeine before an investigation because a participant is not tested in their normal, everyday state. For example, completing a cognitive task might be more difficult for some participants after skipping their habitual caffeine consumption, which could lead to a greater impact on the NVC response. Nevertheless, in basic research investigations, seeking to understand physiological mechanisms/outcomes, it is imperative that caffeine consumption is controlled.

Alcohol has been shown to have a vasodilatory effect, because it stimulates the release of nitric oxide (Tolentino et al., 2011). Furthermore, alcohol is also a known depressant, which will lower the activity of the sympathetic nervous system (Julian et al., 2020). For these reasons, previous work has denoted the importance of controlling alcohol acutely because it leads to an attenuated NVC response (Balogh et al., 2022). The acute consumption of food is more contextual compared with caffeine or alcohol, because it can have both constrictive and dilatory effects. This effect is influenced further by acute food intake, which can modulate autonomic nervous system responses. For example, consuming a meal high in simple sugars will increase parasympathetic nervous system activity owing to a surge in insulin levels (i.e., rest and digest); however, this will ultimately lead to a blood sugar crash eliciting augmented sympathetic nervous system activity (van Baak, 2008). The insulin increase in sympathetic nerve activity is likely to arise through both central mechanisms and peripheral vasodilatation, triggering the arterial baroreflex (McMillan et al., 2022).

As described above, changes in nervous system activity will produce changes in the cerebrovasculature, albeit to a lesser degree than blood pressure or blood gas levels (Smith & Ainslie, 2017). Moreover, given that glucose is the main nutrient used by the brain parenchyma (Mergenthaler et al., 2013), elevations in circulating glucose might, theoretically, alter nutrient delivery/utilization to the working neurons. Nicotine consumed in the form of smoking or vaping will acutely trigger a constrictive response, which can, over time, impair endothelial function (Li et al., 2018). Furthermore, smoking lowers oxygen‐carrying capacity, because haemoglobin has a higher affinity for carbon monoxide compared with oxygen (Sagone et al., 1973). These factors would lead to a less efficient NVC response. Finally, acute exercise has been shown to elicit acute cerebrovascular impairments, which are somewhat dependent on exercise intensity (Burma, Copeland, et al., 2020, Burma, Macaulay, et al., 2020, Burma, Macaulay, Copeland, et al., 2021). Nonetheless, this returns to near baseline levels after recovery, which, over time, will result in long‐term adaptations (Barnes, 2015; Bliss et al., 2021; Erickson et al., 2010). These will be described further below. Although acute exercise has been shown to have a mild effect on the NVC response (Burma, Macaulay, Copeland, et al., 2021; Yamaguchi et al., 2014, Yamaguchi, Ikemura, & Hayashi, 2015, Yamaguchi, Ikemura, Kashima, et al., 2015), it is imperative that researchers should consider this as a confounding factor in their study design.

*Recommendation 3*: Researchers should consider internal/external validity when choosing to restrict caffeine, alcohol, food consumption etc.; at the very least, justification for their decision and time lines for the restrictions (e.g., 2 h for exercise) need to be stated explicitly within the methods sections.

### 4.4 Cardiorespiratory fitness

Regular exercise and physical activity have been shown to have a plethora of physiological benefits, including elevated CBF, angiogenesis, dendritic density, neuroplasticity, vasculature contractility, release of endorphins etc. (Calverley et al., 2020; Lucas et al., 2015). This will lead to reduced stiffness and increased compliancy of the cerebrovasculature; however, Figure 8 highlights very few studies considered the influence of cardiorespiratory fitness on the outcome measures of interest. An elevated training status could, theoretically, lead to improved coupling between metabolic demand and oxygen delivery, in addition to a more efficient extraction rate owing to greater capillary density (Viboolvorakul & Patumraj, 2014). This would, theoretically, reduce the absolute change in blood flow to a given region; however, few NVC investigations have completed a maximal oxygen uptake test concurrently in their samples (Ludyga et al., 2019). Nonetheless, a report on children and adolescents denoted minimal deviations in the NVC response between trained and untrained individuals (Talbot et al., 2023). In addition to aerobic fitness, another consideration is anaerobic fitness levels and/or musculoskeletal strength, because regular weight training can lead to unique physiological adaptations (Smail et al., 2023). However, little is known in this domain, hence more research is warranted. Controlling for cardiorespiratory fitness can be completed through direct quantification (Buttar et al., 2019). If this is not feasible, exercise and physical activity questionnaires could be completed to stratify individuals into different groupings (World Health Organization, 2012).

*Recommendation 4*: Providing a formal assessment of cardiorespiratory fitness in NVC investigations might help to explain the potential variance between groups, because regular exercise and activity improve cerebral and systemic vascular endothelial function.

### 4.5 Controlling for confounding/modifying factors in neuroimaging studies

Control of the above confounding factors can occur in several ways within the study design and/or during statistical analysis (Kestenbaum, 2009; Pourhoseingholi et al., 2012). These work to maximize the internal validity of a study design (Kestenbaum, 2009; Pourhoseingholi et al., 2012). Common approaches to study design consist of restriction, randomization, matching and using a crossover study design (Kestenbaum, 2009; Mills et al., 2009). Restriction occurs when certain factors/individuals are excluded to create a more homogeneous sample. This could be to restrict adolescents to avoid the impact of puberty or to restrict daily habits, such as caffeine, tobacco, exercise etc., as described above (Kestenbaum, 2009). Although this maximizes internal validity, a limitation of this is to reduce the external validity of study findings (as discussed with caffeine) (Kestenbaum, 2009). Researchers could also randomize participants to ensure that confounding variables are evenly distributed between the control and experimental groups (Hariton & Locascio, 2018). However, a more common approach within physiological literature is a randomized crossover design, whereby the same participants complete all arms of a study (Mills et al., 2009). This allows for each participant to act as their own control, which maximizes the internal validity (Mills et al., 2009). A final common way to control for confounding in the study design phase is through matching, whereby participants in control and experimental groups are matched based on relevant factors (e.g., age, sex or concussion history) (de Graaf et al., 2011). Within the statistical analysis, researchers are able to use regression analyses (e.g., analysis of covariance, linear regression or mixed‐effect models) whereby confounding variables are included in the models to adjust the exposure–outcome estimates (e.g., serum hormone analysis) (Kestenbaum, 2009; Pourhoseingholi et al., 2012). Nonetheless, an important consideration is that the number of confounding variables that can be included in a model is dependent on the sample size, whereby a general rule of thumb of one variable per 10 participants is generally the limit of what is recommended (Voorhis & Morgan, 2007). Another option is to stratify the outcome measures into participants who share similar characteristics (Kestenbaum, 2009; Pourhoseingholi et al., 2012). This is recommended in the occurrence of small studies that lack the power to account for these parameters properly in their statistical analysis but provide researchers with an understanding of a given factor that is worthy of further exploration (Kestenbaum, 2009; Pourhoseingholi et al., 2012).

Researchers must carefully complete an a priori sample size calculation based on their data acquisition protocols, data analytical methods and statistical analyses, because these factors could influence the required sample. For example, two studies completing a homogeneous EEG–fMRI assessment of a visual task might differ in their sample size requirements if one derives EEG microstates and uses analysis of covariance to discern differences between groups, whereas the other uses time–frequency analysis with a cross‐over design and linear mixed‐effect models (Mills et al., 2009). Likewise, a more stimulating/activating task might elicit a greater difference between a clinical and healthy presentation and thus would require fewer participants to achieve a meaningful difference (Roby et al., 2024). Therefore, a one‐size‐fits‐all approach to determine the minimum sample size for the field of NVC is an oversimplification.

*Recommendation 5*: Consideration of known confounding factors needs to be accounted for to minimize these factors within the study design and/or by collecting extra information to be included within the planned statistical analysis.

### 4.6 Clinical relevance

Figure 6 highlights that 56 (15.4%) articles were completed in clinical populations to ascertain differences in comparison to their healthy counterparts. The discussion of controlling for confounding influences has important clinical translational implications, particularly for understanding cerebrovascular and neurological diseases. Impairments in NVC have been linked to a wide range of conditions (e.g., stroke, cognitive decline, Alzheimer's disease and traumatic brain injury). However, in comparison to healthy control subjects, patients are likely to have additional comorbidities that can prove equally complex (e.g., impaired local and systemic vascular endothelial function or advanced atherosclerosis), and thus a higher resting blood pressure. Certain clinical populations might experience a greater degree of grey matter atrophy and reduced myelination, altering the transmission between neurons, notwithstanding structural and volumetric changes to the neurovascular unit. Therefore, although NVC differences might be more prominent in clinical populations, these could, nonetheless, be moderated by changes in blood pressure and/or $P_{\mathrm{ET},CO_{2}}$. Moreover, failure to control for lifestyle factors and body habits could confound the outcomes obtained. For example, exercise has been demonstrated to attenuate the NVC response acutely, whereby individuals in a healthy cohort who are tested immediately after exercise might display results similar to a clinical comparative group. Hence, the use of a rigorous study design will maximize the power within a given study to ascertain clinical NVC differences.

### 4.7 Limitations

A common limitation found within systematic reviews is publication bias, whereby studies that present significant findings are more likely to be published (Nair, 2019). Although this bias is generally the most apparent within clinically based systematic reviews, it cannot be ruled out that this did not occur here. For example, research laboratories have attempted to perform multimodal imaging protocols but were unsuccessful owing to different constraints (e.g., lack of expertise, equipment or data‐processing capabilities). Furthermore, a few articles were not able to be retrieved, with only articles written in English being included in this review. Nevertheless, the primary purpose of the present review was to examine the physiological control within published multimodal studies, and the vast majority received a poor risk‐of‐bias rating (>90%). Thus, despite missing a few articles and the potential for a publication bias, this would have a nominal impact, given that the main finding of the present review is the need for better control in future studies.

Based on the heterogeneous methodological approaches, tasks used, equipment used and outcomes of the included studies, it was not feasible to complete a meta‐analysis or pooled estimates for any outcome metrics of interest. For example, previous studies have highlighted that different fMRI scanners will result in divergent resting‐state metrics (Kayvanrad et al., 2021). Additionally, despite the use of a similar fMRI sequence, studies might have used different tasks (e.g., visual or cognitive) and/or had heterogeneous EEG electrode placement. These factors led to the present review adopting a narrative approach; however, future studies should seek to maximize homogeneity with published studies. Finally, given that the present review included a plethora of neuroimaging devices, the potential for conflicts of interest from given neuroimaging companies is likely. With the surge in the number of commercial companies producing neuroimaging equipment, this will occur increasingly over the coming years. Thus, it is imperative that manuscripts openly declare potential conflicts of interest, especially if the authors are co‐founders/owners of a company.

A paucity of investigations has focused on potential race and/or ethnicity differences in cerebrovascular and neurological imaging literature (Wallace et al., 2023). Emerging research is demonstrating that some neuroimaging equipment can be influenced by the amount of melanin within the skin (Kwasa et al., 2023). Moreover, health disparities and the prevalence of various conditions (e.g., hypertension, stroke, Alzheimer's disease and related dementias) differ based on biological differences between races/ethnicities. Finally, considering race/ethnicity in neuroimaging will help to minimize bias within machine learning algorithms, ensuring that artificial intelligence‐driven tools are generalizable across diverse populations (Wang et al., 2023).

## 5 CONCLUSIONS

The present systematic review summarized the existing literature that has used a multimodal approach to quantify cerebrovascular haemodynamics and neuronal function concurrently. Although heterogeneous approaches were taken, the majority of studies displayed inadequate and/or complete lack of control of physiological influences, daily confounding factors, menstrual cycle or cardiorespiratory fitness status. To understand the NVC response fully, it is imperative to remove physiological noise to ensure that a recorded signal is the result specifically of neuronal activity and not other systemic influences. This is especially paramount within clinical populations, in order to maximize the sensitivity of determining differences relative to healthy control subjects. Ultimately, the present review highlights the feasibility and utility of conducting multimodal assessments; however, it is a necessity for researchers to implement strategies to maximize control for confounding factors and take them into account for their data interpretation/dissemination.

## AUTHOR CONTRIBUTIONS

Joel S. Burma: Conceptualization; methodology; validation; formal analysis investigation; writing—original draft; writing—review and editing; visualization. Damian M. Bailey: Methodology; writing—review and editing. Nathan E. Johnson: Validation; formal analysis; investigation; writing—review and editing. James K. Griffiths: Validation; formal analysis; investigation; writing—review and editing. Josh J. Burkart: Validation; formal analysis; investigation; writing—review and editing. Clara A. Soligon: Validation; formal analysis; investigation; writing—review and editing. Elizabeth K.S. Fletcher: Validation; formal analysis; investigation; writing—review and editing. Raelyn M. Javra: Validation; formal analysis; investigation; writing—review and editing. Chantel T. Debert: Conceptualization; methodology; writing—review and editing. Kathryn J. Schneider: Conceptualization; methodology; writing—review and editing. Jeff F. Dunn: Conceptualization; methodology; writing—review and editing. Jonathan D. Smirl: Conceptualization; methodology; writing—review and editing.

## CONFLICT OF INTEREST

Damian M. Bailey is Editor‐in‐Chief of *Experimental Physiology*, Chair of the Life Sciences Working Group, a member of the Human Spaceflight and Exploration Science Advisory Committee to the European Space Agency and a member of the Space Exploration Advisory Committee to the UK Space Agency. Damian M. Bailey is also affiliated to Bexorg, Inc. (USA) focused on the technological development of novel biomarkers of cerebral bioenergetic function and structural damage in humans.

## Data Availability

Data from the systematic review are available upon reasonable request to the lead author (Joel S. Burma). This includes supplemental material consisting of the search strategy, full bibliography, risk‐of‐bias scoring, sex and age of included subjects, neuroimaging equipment and tasks used by each study, and a list of all clinical studies.
